# Effect of a text message intervention on alcohol-related harms and behaviours: secondary outcomes of a randomised controlled trial

**DOI:** 10.1186/s13104-019-4308-y

**Published:** 2019-05-14

**Authors:** Sarah Sharpe, Bridget Kool, Robyn Whittaker, Arier C. Lee, Papaarangi Reid, Ian Civil, Shanthi Ameratunga

**Affiliations:** 1 Section of Epidemiology and Biostatistics, School of Population Health, Faculty of Medical & Health Sciences, University of Auckland, Private Bag 92019, Auckland, 1142 New Zealand; 2 National Institute for Health Innovation, University of Auckland, and Waitemata District Health Board, Auckland, New Zealand; 3 Te Kupenga Hauora Māori, Faculty of Medical & Health Sciences, University of Auckland, Auckland, New Zealand; 4 Trauma Service, Auckland City Hospital, Auckland, New Zealand

**Keywords:** Alcohol drinking, Injuries, Cell phones, Text messaging, mHealth intervention, brief intervention for harm (BI)

## Abstract

**Objective:**

Mobile Health approaches show promise as a delivery mode for alcohol screening and brief intervention. The ‘YourCall’ trial evaluated the effect of a low-intensity mobile phone text message brief intervention compared with usual care on hazardous drinking and alcohol-related harms among injured adults. This paper extends our previously published primary outcome analysis which revealed a significant reduction in hazardous drinking associated with the intervention at 3 months, with the effect maintained across 12 months follow-up. The objective of the current study was to evaluate the effect of the intervention on alcohol-related harms and troubles and help-seeking behaviours (secondary outcomes) at 12-months follow-up.

**Results:**

A parallel two-group, single-blind, randomised controlled trial was conducted in 598 injured inpatients aged 16–69 years identified as having medium-risk hazardous drinking. Logistic regression models applied to 12-month follow-up data showed no significant differences between intervention and control groups in self-reported alcohol-related harms and troubles and help-seeking behaviours. Although this text message intervention led to a significant reduction in hazardous alcohol consumption (previously published primary outcome), changes in self-reported alcohol-related harms and troubles and help seeking behaviours at 12-months follow up (secondary outcomes) were small and non-significant.

**Trial registration:**

ACTRN12612001220853. Retrospectively registered 19 November 2012.

## Introduction

Alcohol-related harms are a significant global issue, causing large health, social and economic burdens to people, communities, and society [1–3]. An individual’s hazardous drinking can cause a wide range of harms (e.g. physical, mental, relationship, employment, financial and legal) to themselves as well as others [4, 5].

Alcohol Screening and Brief Interventions (SBI) can reduce alcohol consumption and alcohol-related harms [6–8], and digital (including mobile health–mHealth) approaches could enhance the scalability of such interventions [9]. As described elsewhere [10], our study group developed a low-intensity, automated, culturally-appropriate, brief text message intervention (called ‘YourCall’), drawing on SBI principles [11] and Stages of Change behaviour change theory [12], and designed to reduce hazardous drinking and alcohol-related harm among adults admitted to hospital following an injury. A randomised controlled trial (RCT) evaluating the intervention found a significant effect in the primary outcome, i.e., a reduction in hazardous drinking in the intervention group compared with control (usual care) group [13]. The effect was maintained across follow-up points (three, six and 12 months) and was similar among Māori (New Zealand’s indigenous population) and non-Māori, and among younger (16–29 years) and older (30–69 years) participants.

This paper extends our previously published RCT findings, by evaluating secondary outcomes from the RCT’s 12-month follow-up survey, which investigated the effects of the ‘YourCall’ text message intervention on a range of self-reported alcohol-related harms and troubles and participants’ alcohol-related help-seeking behaviours. The hypothesis of this study was that, in comparison to hazardous drinkers discharged from hospital following an injury admission who received usual care, those receiving the intervention would have experienced less alcohol-related harms at the 12-month follow-up point.

## Main text

### Methods

#### Study design and participants

The RCT design, patient population and eligibility criteria are described in the published study protocol [14] and the paper reporting the primary outcome [13]. A two-group, parallel, RCT conducted in Auckland, New Zealand, in 598 trauma inpatients with medium-risk hazardous drinking patterns, compared the effects of the ‘YourCall’ text message intervention with ‘usual care’ on hazardous drinking and alcohol-related harms. The trial only included patients with medium-risk drinking patterns as BIs are treatments designed specifically for medium-risk drinkers rather than drinkers at higher risk of harm and dependent on alcohol, for whom the appropriate management involves counselling, specialist evaluation and treatment [11]. The trial was approved by the New Zealand Health and Disability Ethics Committee (12/NTB/28), registered with the Australian New Zealand Clinical Trials Registry (anzctr.org.au; Identifier: ACTRN12612001220853), and followed the Consolidated Standards of Reporting Trials (CONSORT) guidelines [15]. Written informed consent was obtained from all participants.

#### Procedures

The group randomised to the intervention received 16 text messages over four weeks, commencing 7–10 days after hospital discharge. Control group participants received one text message acknowledging their participation and indicating they would be contacted in three months’ time.

Participants’ baseline assessments included demographic data and responses to the Alcohol Use Disorders Identification Test (AUDIT). Participants were invited to complete the AUDIT-C via text message at 3- and 6-months follow-up and complete a web-based survey at 12 months which included the AUDIT and other alcohol-related questions (described in more detail next). Participants who did not respond to the web survey were contacted by research assistants to complete assessments via telephone.

#### Secondary outcome measures assessed at 12-months follow-up

Alcohol-related harms and troubles were assessed using the ‘Alcohol Harms’ and ‘Alcohol Troubles’ seven-item checklists, drawn from the Gender, Alcohol, and Culture International Study (http://www.genacis.org./11) [16, 17]. All questions had a possible value 0–2, and the total score for each checklist ranged between 0 and 14.

Help-seeking behaviour was assessed by asking participants if they did any of the following: (a) rang the Alcohol Drug Helpline (offers free confidential professional advice); (b) visited the Alcohol Drug Helpline website; (c) visited other websites for information or help relating to alcohol use; (d) talked with a doctor or other health professional about their drinking; (e) talked with anyone else, such as friends or family, about their or others’ drinking. ‘Behaviours a–d’ (reflecting professional sources of help) were assessed as a composite outcome called ‘Help-seeking behaviours 1’. ‘Behaviour e’ was assessed separately as informal sources, referred to as ‘Help-seeking behaviour 2’ in this analysis.

## Statistical analysis

Data were analysed following a pre-specified analysis plan. Baseline demographic variables (age, sex, and ethnic group), employment, and AUDIT-C mean scores, and 12-month survey question responses were summarised for the intervention and control groups.

The differences between the intervention and control groups in secondary outcome measures were analysed using logistic regression models adjusted for the randomisation variables of age, sex, hospital centre, ethnicity, and baseline AUDIT-C score.

Data analyses were performed using SAS version 9.4 (SAS Institute Inc. Cary NC). All statistical tests were two-tailed. All evaluations were performed on the ‘intention to treat’ principle, no adjustments were made for multiplicity of any outcomes, and no imputations were made for missing data.

## Results

**Fig. 1:**
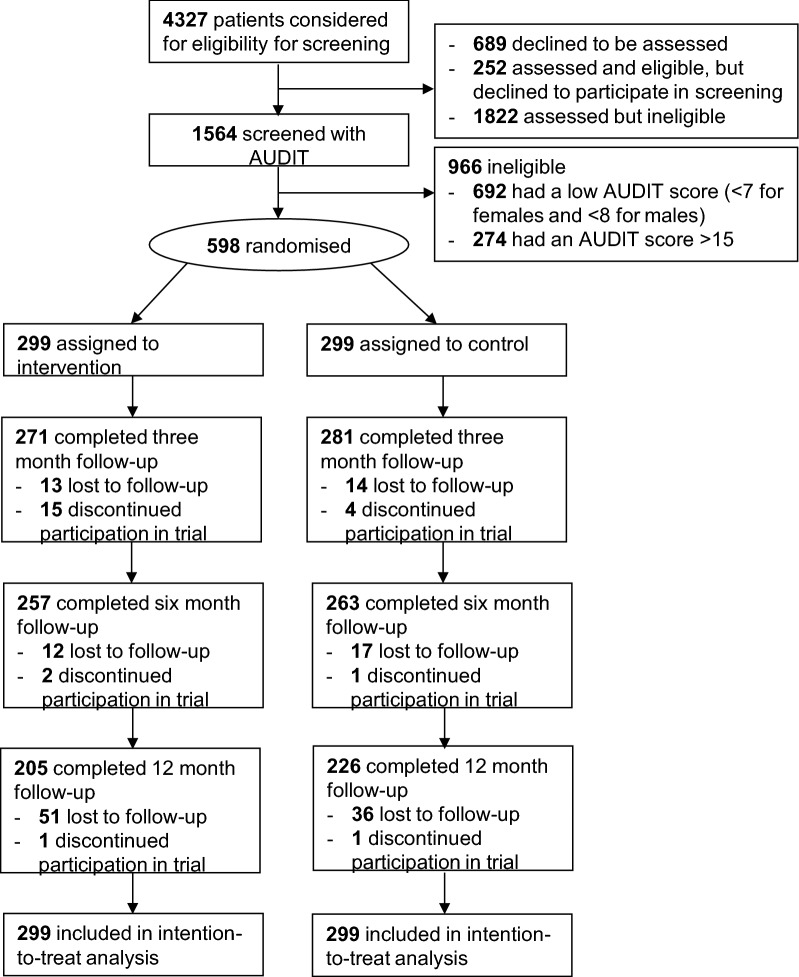
CONSORT flow diagram for YourCall trial

As previously described [13], 598 of the 1564 potentially eligible participants who were screened met the trial inclusion criteria (Fig. 1). The characteristics of the two groups were similar at baseline, including mean AUDIT-C scores (control group: 6.82 [95% CI 6.62–7.03]; intervention group: 6.87 [95% CI 6.68–7.06]). Twelve-month follow-up data were provided by 226 (76%) of the 299 control group participants and 205 (69%) of the 299 intervention group participants. The percentages of females and Māori respondents at 12 months were similar to those at baseline, however there were fewer participants in the 16–29-year-old group at 12 months compared to baseline (Table 1).

**Table 1:** Characteristics of participants at baseline and 12-months

Characteristics	Baseline		12-month follow-up point	
	Control group n = 299 n (%)	Intervention group n = 299 n (%)	Control group n = 226 n (%)	Intervention group n = 205 n (%)
Female	86 (28.8)	85 (28.4)	67 (29.6)	63 (30.7)
Age (mean, SD)	34 (13)	34 (13)	36 (13)	35 (13)
Age group 16–29 yrs	144 (48.2)	145 (48.5)	90 (39.8)	94 (45.9)
Māori ethnicity	64 (21.4)	62 (20.7)	43 (19.0)	36 (17.6)

The group specific responses relating to alcohol harms, alcohol troubles, and help seeking are presented next, followed by the results of multivariable regression models which indicate that the intervention did not have a significant effect on these secondary outcomes.

Among participants responding to the ‘alcohol harms’ questions in the 12-month survey (Table 2), over half (124 [55%] of the control group and 106 [53%] of the intervention group) reported alcohol-related harmful effects in one or more of the following domains: work, studies, or employment; housework or chores around the house; marriage/intimate relationships; relationships with other family members including children; friendships or social life; finances; physical health. Of those responding to the ‘alcohol troubles’ questions, one-third of both control and intervention groups (76 [34%] and 65 [33%], respectively) reported alcohol-related troubles in one or more of the following domains: the law; illness connected with drinking; losing or nearly losing a job; feeling annoyed by other people’s criticisms of their drinking; having a spouse or someone close leave or threaten to leave; loss of a friendship; getting into a fight.

**Table 2:** Summary of responses to 12-month survey

12-month survey domains and questions	Control group n = 226 n (%)	Intervention group n = 205 n (%)
*Alcohol harms* Reports a harmful effect during the past 12 months from drinking alcohol^a^	*n *= *225* 124 (55.1)	*n *= *199* 106 (53.3)
*Alcohol troubles* Reports experiencing trouble during the past 12 months from drinking alcohol^b^	*n *= *223* 76 (34.1)	*n *= *200* 65 (32.5)
*Help-seeking behaviours 1* Reports any of the following behaviours in the last 12 months: a) ringing Alcohol Helpline, b) looking at Alcohol Helpline website, c) looking at other website for information or help about alcohol, d) talking with a doctor or other health professional about respondent’s drinking	*n *= *219* 24 (11.0)	*n *= *194* 31 (16.0)
*Help-seeking behaviour 2* Reports having talked in the last 12 months with someone else, such as friends or family about the respondent or someone else’s drinking	*n *= *222* 90 (40.5)	*n *= *197* 85 (43.2)

^a^Alcohol Harms score of 1 or more (i.e. score of 0 indicates no harm, and a score of 1 or more indicates one or more harms)

^b^Alcohol Troubles score of 1 or more (i.e. score of 0 indicates no troubles, and a score of 1 or more indicates one or more troubles)

A small number of respondents (24 [11%] of the control group and 31 [16%] of intervention group) had sought help in relation to their drinking through contacting a health professional, via the Alcohol Drug Helpline, or through seeking information or help from online sources (‘help-seeking behaviours 1’ in Table 2). In contrast, 90 (41%) control group respondents and 85 (43%) intervention group respondents reported talking with others, such as friends or family, about their drinking (‘help-seeking behaviour 2’ in Table 2).

The logistic regression models adjusted for age, sex, hospital, ethnicity, and baseline AUDIT-C score found that there were only small and non-significant differences between intervention and control groups in relation to self-reported alcohol-related harms (OR 0.88, 95% CI 0.60–1.30, p = 0.53) and alcohol-related troubles (OR 0.89, 95% CI 0.59–1.35, p = 0.58). The evidence of an intervention effect on increased help seeking in relation to their alcohol use from professional and informal sources was also weak (‘help-seeking behaviours 1’: OR 1.67, 95% CI 0.93–3.01, p = 0.09; and ‘help-seeking behaviours 2’: OR 1.16, 95% CI 0.78–1.72, 0.48).

## Discussion

This study reports the analysis of secondary outcome data collected at 12-months follow-up in an RCT evaluating the effect of a low-intensity mobile phone text message intervention compared with usual care on hazardous drinking and alcohol-related harms among injured adults. While the primary outcome analysis found a significant reduction in hazardous drinking in the intervention group compared with control (usual care) group, we did not detect any important differences between treatment groups in the measures of alcohol-related harms and troubles and help-seeking behaviours at 12-months follow-up.

The strengths of this trial include the trial’s size, broad age range, generalisability to adult inpatient trauma care patients, focus on medium-risk drinkers, and recruitment practices that ensured a high participation of Māori patients. The study was not limited to patients wanting to change their drinking or wanting help. Many SBI trials only measure alcohol consumption outcomes. This trial included alcohol-related harms and troubles and help-seeking behaviours as secondary outcomes.

The findings described in this paper are similar to other trials evaluating the effect of alcohol SBI on alcohol-related negative consequences which have reported no differences between treatment groups [18, 19], yet contrast with other studies which have reported significant differences [6, 20]. In SBI trials that report on alcohol-related harms, the measures and outcomes are wide-ranging and difficult to compare across studies [7, 9]. Few SBI trials have evaluated alcohol-related help-seeking behaviours and the findings have been inconsistent [18, 21]. Neumann et al. [21] reported more frequent use of community alcohol treatment services by ‘at-risk drinking’ injured patients who received computer-generated BI in the ED compared with control group participants who received screening only, during a one year follow-up period. In contrast, D’Onofrio et al. [18] found that the rate of health service utilisation did not differ by treatment group in their study of staff-administered BI compared to scripted discharge instructions.

There are several possible explanations for the null findings of this study. The content of the text messages focussed mainly on reducing alcohol consumption. This may explain the stronger effects of this intervention on hazardous alcohol use (primary outcome [13]) and the weak effects on alcohol-related harm and help seeking. Furthermore, as the study was powered for the primary outcome and not the outcomes examined in the present analysis, Type II error could account for the weak treatment effects observed. The baseline assessment, screening, and repeated administration of the AUDIT-C at follow-up could have also acted as a form of treatment for the control group, creating a beneficial effect and decreasing differences in secondary outcome measures between the intervention and control groups [19, 22]. Factors outside the scope of the intervention such as inequities in access to information and treatment services, and perceptions of social norms and stigmatisation, could also influence the likelihood of help seeking—although we have no reason to assume these were differentially distributed in the intervention and control groups in our trial.

In conclusion, although mHealth BIs are promising strategies for helping people to reduce their alcohol consumption and change their drinking patterns, our findings raise questions about the role of mHealth BI in reducing alcohol-related harms. Further research is required to investigate if enhanced programme content on harms and sources of support for self-management and treatment could strengthen the effectiveness of mHealth interventions. Regardless of the potential benefits that could accrue, BIs should be viewed as a health sector strategy that is one component of a multi-pronged public health approach. Alcohol-related harms are inequitably distributed at a societal level and mediated by complex and multi-factorial pathways including pervasive commercial determinants of health [3, 23, 24]. Consequently, addressing the price, availability, advertising and marketing of alcohol should remain cornerstones of equity-focused harm reduction strategies [23, 25].

## Limitations

As secondary outcome measures were only assessed at 12 months due to concerns regarding the length of the baseline assessment and potential treatment effects in the control group [22, 26], we cannot assess if there were differential changes in these measures from baseline to 12 months. As evident in our primary outcome analysis [13] focusing on differences in hazardous drinking in the intervention and control groups (measured using the AUDIT-C), mean AUDIT-C scores were similar in both groups at baseline. Although not assessed directly, both groups are also likely to have had similar baseline scores in measures of alcohol-related harms and troubles given the randomisation procedure. The notable finding from our primary outcome analysis was evidence of an important (statistically significant) intervention effect, over and above observed declines in mean AUDIT-C scores over the follow-up period in both the intervention and control groups. While this trial was not designed to investigate changes in alcohol-related harms and troubles over time, the statistically non-significant differences between the two groups with respect to these measures in the 12-month survey suggest that evidence of any intervention effect on these secondary outcomes was weak. However, a Type II error could have a stronger influence in these analyses as the study was only powered for the primary outcome.

Other limitations include follow-up rates at 12 months that were lower than those at three and six months and differed between the two treatment groups, and self-reported measures that could be susceptible to measurement bias [27]. While the latter could result in inaccuracies such as under-reporting, this is unlikely to differ between the randomised treatment groups.

## Data Availability

Due to the conditions of the informed consent obtained from participants, the institutional and Ministry of Health ethical requirements do not permit us to share participant data from this study.

## Abbreviations

AUDIT: alcohol use disorders identification test
AUDIT-C: alcohol use disorders identification test—consumption
BI: brief intervention
CI: confidence interval
CONSORT: consolidated standards of reporting trials
ED: emergency department
mHealth: mobile health
OR: odds ratio
RCT: randomised controlled trial
SBI: screening and brief intervention
